# Association of complication of type 2 diabetes mellitus with hemodynamics and exercise capacity in patients with heart failure with preserved ejection fraction: a case–control study in individuals aged 65–80 years

**DOI:** 10.1186/s12933-023-01835-2

**Published:** 2023-04-28

**Authors:** Yousuke Sugita, Katsuhiko Ito, Yui Yoshioka, Satoshi Sakai

**Affiliations:** 1 Faculty of Health Sciences, Tsukuba University of Technology, 4-12-7, Kasuga, Tsukuba, Ibaraki 305-8521 Japan; 2 Department of Rehabilitation, National Hospital Organization Matsumoto National Hospital, Matsumoto, Japan; 3 Department of Rehabilitation, Musashino General Hospital, Kawagoe, Japan

**Keywords:** Heart failure with preserved ejection fraction, Type 2 diabetes mellitus, Peak oxygen uptake, Peak stroke volume, Peak heart rate, Peak arteriovenous oxygen difference, Ventilatory equivalent versus carbon dioxide output slope, Anemia, Sarcopenia, Echocardiography

## Abstract

**Background:**

Type 2 diabetes mellitus (T2DM) is a frequently observed complication in patients with heart failure with preserved ejection fraction (HFpEF). Although a characteristic finding in such patients is a decrease in objective exercise capacity represented by peak oxygen uptake (peakVO_2_), exercise capacity and its predictors in HFpEF with T2DM remain not clearly understood. This case–control study aimed to investigate the association between exercise capacity and hemodynamics indicators and T2DM comorbidity in patients with HFpEF aged 65–80 years.

**Methods:**

Ninety-nine stable outpatients with HFpEF and 50 age-and-sex-matched controls were enrolled. Patients with HFpEF were classified as HFpEF with T2DM (n = 51, median age, 76 years) or without T2DM (n = 48, median age, 76 years). The peakVO_2_ and ventilatory equivalent versus carbon dioxide output slope (VE vs VCO_2_ slope) were measured by cardiopulmonary exercise testing. The peak heart rate (HR) and peak stroke volume index (SI) were measured using impedance cardiography, and the estimated arteriovenous oxygen difference (peak a-vO_2_ diff) was calculated with Fick's equation. The obtained data were compared among the three groups using analysis of covariance adjusted for the β-blocker medication, presence or absence of sarcopenia, and hemoglobin levels in order to determine the T2DM effects on exercise capacity and hemodynamics in patients with HFpEF.

**Results:**

In HFpEF with T2DM compared with HFpEF without T2DM and the controls, the prevalence of sarcopenia, chronotropic incompetence, and anemia were significantly higher (p < 0.001). The peakVO_2_ (Controls 23.5 vs. without T2DM 15.1 vs. with T2DM 11.6 mL/min/kg), peak HR (Controls 164 vs. without T2DM 132 vs. with T2DM 120 bpm/min), peak a-vO_2_ (Controls 13.1 vs without T2DM 10.6 vs with T2DM 8.9 mL/100 mL), and VE vs VCO_2_ slope (Controls 33.2 vs without T2DM 35.0 vs with T2DM 38.2) were significantly worsened in patients with HFpEF with T2DM (median, p < 0.001). There was no significant difference in peak SI among the three groups.

**Conclusions:**

Our results suggested that comorbid T2DM in patients with HFpEF may reduce exercise capacity, HR response, peripheral oxygen extraction, and ventilation efficiency. These results may help identify cardiovascular phenotypes of HFpEF complicated with T2DM and intervention targets for improving exercise intolerance.

**Supplementary Information:**

The online version contains supplementary material available at 10.1186/s12933-023-01835-2.

## Background

Heart failure (HF) cases have been increasing worldwide, and the number of patients with HF is estimated to be 26 million [1–3]. In addition, type 2 diabetes mellitus (T2DM) is a global epidemic, with a continuous rise in the number of patients yearly [4]. Among patients with HF, HF with preserved ejection fraction (HFpEF) accounts for approximately 50% [5]. One-third of patients with HFpEF have DM-related complications [6], which are associated with high hospitalization rates and poor life prognosis [7, 8]. Therefore, elucidating the cardiovascular phenotype of patients with HFpEF with T2DM may help identify intervention targets.

Exercise intolerance, such as decreased peak oxygen uptake (peakVO_2_) objectively measured by cardiopulmonary exercise testing, is a common clinical symptom of HFpEF and T2DM [9, 10]. In addition, patients with HFpEF with DM have significantly reduced exercise capacity compared to those with HFpEF without DM [11], and exercise intolerance in patients with DM is one of the vital determinants of life prognosis [12]. However, the underlying cause of exercise intolerance in patients with HFpEF with DM remains unclear. A previous study [13] reported that patients with T2DM had left ventricular [LV] structural and functional abnormalities from the asymptomatic stage, and as the number of LV defects increased, the peakVO_2_ decreased. The results of this study suggested that a decreased central hemodynamic response may be associated with peakVO_2_ in patients with T2DM, but the daily physical activity or peripheral oxygen extraction capacity was not measured. In Fick's formula, oxygen uptake is determined by central factors, such as cardiac output (CO), and peripheral oxygen extraction capacity, such as arteriovenous oxygen difference (a-vO_2_ diff). The causes of exercise intolerance in patients with HFpEF are thought to be both central factors due to decreased CO [14] and peripheral factors due to decreased arteriovenous oxygen difference [9]. However, the causes of patients with HFpEF with DM, including hemodynamics during submaximal exercise and peripheral tissues, such as sarcopenia, have not been comprehensively investigated.

Therefore, we hypothesized that patients with HFpEF with T2DM had a lower exercise capacity and reduced central hemodynamics response during submaximal exercise compared to those with HFpEF without T2DM and age- and sex-matched control. This case–control study aimed to investigate the association between hemodynamic response and exercise capacity and complication with T2DM in patients with HFpEF aged 65–80 years.

## Methods

### Study design and participants

Ninety-nine patients with HFpEF and 50 age-and sex-matched controls were prospectively enrolled from April 2016 till March 2020. All patients were outpatients with stable symptoms and classified into two groups according to the presence or absence of T2DM. In addition to patients with HFpEF, we recruited a control group of 50 individuals without cardiovascular disease and interventions. The final analysis included 50 individuals in the control group, 48 in the HFpEF-without-T2DM group, and 51 in the HFpEF-with-T2DM group. Details of the study protocol and diagnostic criteria for HFpEF and T2DM [15–18] are described in Additional file 1. All patients with HFpEF had New York Heart Association (NYHA) functional classification II or III.

All participants provided written informed consent. This study was conducted in accordance with the tenets of the Declaration of Helsinki, and the study protocol was approved by the Institutional Review Board of Tsukuba University of Technology in Tsukuba City, Japan (Approval Number: 202108).

### Anthropometric parameters, biochemical analysis, and blood pressure

The body mass index (BMI) and body surface area (BSA) were calculated by measuring height and weight (Additional file 1). Overweight and obesity were determined from the calculated BMI based on the World Health Organization (WHO) criteria for obesity [19]. The BSA was calculated using Dubois et al.'s formula (Additional file 1) [20].

Blood was drawn from study participants after 12 h of fasting and before ingesting medications. After collecting 10 mL of blood, the brain natriuretic peptide (BNP), triglyceride, total cholesterol, high-density lipoprotein cholesterol, low-density lipoprotein cholesterol, hemoglobin A1c, hemoglobin, fasting plasma glucose, plasma glucose, and insulin levels were measured (Additional file 1).

We also calculated the homeostasis model assessment of insulin resistance [21] and the estimated glomerular filtration rate [22] (Additional file 1). Anemia was defined as a hemoglobin level of < 13 g/dL in men and < 12 g/dL in women (WHO criteria) [23].

Systolic and diastolic blood pressures were measured from the arms of seated participants after a 20 min rest using an automatic blood pressure monitor (HEM-7220, Omron Healthcare Co., Ltd. Kyoto, Japan). Hypertension and dyslipidemia were diagnosed according to the Japanese diagnostic criteria (Additional file 1) [24].

### Echocardiography

Structural and functional abnormalities of the LV and left atrium (LA) were assessed using echocardiography (ACUSON SC2000; 4V1c, and 4Z1c probes; Siemens Japan K.K. Tokyo, Japan) with individuals in the left decubitus position. The LV posterior wall thickness at end-diastole, interventricular septal thickness at end-diastole, LV end-diastolic diameter, LV end-systolic diameter, LV diameter, and LV wall thickness were recorded in M-mode. The LV end-diastolic and end-systolic volumes were measured using the biplane-modified Simpson method. The relative wall thickness (RWT) and LV myocardial weight were calculated using Devereux's formula [25]. Formulas for the calculation of LVEF and stroke volume (SV) are shown in Additional file 1.

LV inflow parameters were obtained using pulse-wave tissue doppler in the apical four-chamber view. The peak early flow velocity, late diastolic flow velocity, ratio of peak early and late diastolic flow velocities, and early diastolic flow wave deceleration time were assessed. Pulsed-wave tissue doppler was conducted to obtain the peak early diastolic tissue velocity at the septal and lateral aspects of the mitral annulus. The mitral inflow early diastolic velocity ratio to the average velocity from the septal and lateral sides of the mitral annulus was calculated to estimate the LV filling pressure. The pulmonary artery systolic pressure was estimated according to the methods presented in Additional file 1 [26]. In addition, a detailed evaluation of mitral regurgitation (MR) and its severity was also presented in Additional file 1 [27, 28].

Based on the report of Lang et al. [29], LV hypertrophy (LVH) was defined as an LV mass index > 115 g/m^2^ for men and > 95 g/m^2^ for women. LV concentric remodeling was defined as LVH (−) and an RWT > 0.42; LV eccentric hypertrophy was defined as LVH ( +) and an RWT < 0.42; and LV concentric hypertrophy was defined as LVH ( +) and an RWT > 0.42.

The LA volume (LAV) was measured in three different sequences of the cardiac cycle. The maximum LAV was measured just before the mitral valve opened, and the pre-A LAV (before atrial contraction) was determined at the onset of atrial contraction (P-wave peak electrocardiogram), while the minimum LAV was measured when the mitral valve was closed. All volumes were determined according to the biplane method in four and two-chamber views. The LA emptying fraction, the comprehensive reservoir function of LA, was calculated using the formula shown in Additional file 1. The LAV index was calculated using the methods and formulas shown in Additional file 1 [30].

### Speckle-tracking imaging

LV myocardial deformation was assessed using the two-dimensional speckle-tracking technique in three apical views at a temporal resolution of 60–90 frames/s (Additional file 1). The LV global longitudinal strain (LV-GLS) represented LV shortening in the longitudinal plane [31]. Furthermore, LA speckle-tracking imaging, longitudinal strain, and strain rate curves were generated for each of the six atrial segments obtained from apical four-chamber and two-chamber views. The peak LA strain (LA-GLS) was calculated by averaging each value observed in all six LA segments analyzed [32].

### Measurement of the epicardial adipose tissue thickness

For epicardial adipose tissue thickness measurements, all participants underwent echocardiography, as proposed by Iacobellis et al. (Additional file 1) [33].

### Measurement of exercise capacity and hemodynamic response

Exercise capacity was measured using cardiopulmonary exercise testing (CPET) with a symptomatic limit using an ergometer (232C-XL; Combi Co., Ltd., Tokyo, Japan). The peakVO_2_ [34], work rate at peak exercise (peak watt), anaerobic threshold (ATVO_2_) [35], and work rate at AT exercise (AT watt) were measured according to the methods in Additional file 1. The ventilatory equivalent versus carbon dioxide output slope (VE vs VCO_2_ slope) was measured by selecting a range from the point at which VE began to increase during ramp loading to the respiratory compensation point. Heart rate recovery (HRR) and oxygen pulse were calculated using the methods presented in Additional file 1.

The hemodynamic response from sitting to peak exercise was measured using a noninvasive transthoracic bioimpedance device (PhysioFlow PF-05 Lab1; Manatec Biomedical, Paris, France) during CPET. The measurement items in PhysioFlow were SV and HR. The stroke volume index (SI), cardiac output index (CI), and arteriovenous oxygen difference (a-vO_2_ diff) values were calculated using the methods in Additional file 1. Chronotropic incompetence and an abnormal HRR value were determined using the methods shown in Additional file 1 [36, 37].

### Measurement of physical activity

Daily physical activity was estimated from the magnitude and frequency of the acceleration signal detected at 32 Hz using a pedometer with a multiple memory accelerometer (Lifecorder; SUZUKEN CO., LTD. Nagoya, Japan). We assumed a step count value of  > 20,000 steps/day and < 500 steps/day were not routine step count values [38]. Detailed measurement methods are described in Additional file 1.

### Diagnosis of sarcopenia

Sarcopenia was defined according to the Asian Working Group for Sarcopenia 2019 [39]: a skeletal muscle mass index of < 7.0 kg/m^2^ for men and < 5.7 kg/m^2^ for women; a grip strength of  < 28 kg for men and < 18 kg for women; or a five-time chair-stand test time ≥ 12 s. The skeletal muscle mass index, grip strength, and five-time chair-stand test were measured as described in Additional file 1.

### Statistical analysis

Normally distributed data are expressed as means ± standard deviations, whereas non-normally distributed data are expressed as medians, and nominal data are expressed as percentages. SPSS version 29 (IBM Japan, Ltd. Tokyo, Japan) was used for all the statistical analyses. The significance level was set to P < 0.05 using a two-tailed test. For data analysis, we tested the normality using the Shapiro–Wilk test. One-way analysis of variance and the Tukey post hoc test was conducted for normally distributed variables, and the Kruskal–Wallis test with Bonferroni correction was conducted for non-normally distributed variables. The *χ*^2^ test with Bonferroni correction was carried out for nominal-scale data. One-way analysis of variance, *χ*^2^ test, and Kruskal–Wallis test were used to compare the differences in all data between the three groups. All groups were compared for exercise capacity, work rate, and hemodynamics using analysis of covariance adjusted for the β-blocker medication, presence or absence of sarcopenia and hemoglobin levels. Furthermore, to examine the effects of T2DM complication on the exercise capacity of patients with HFpEF, stepwise method multiple linear regression analysis was performed to investigate the independent association between peakVO_2_ and T2DM in Japanese patients with HFpEF. A multiple linear regression analysis with peakVO_2_ as the dependent variable was performed, while the independent variables included age [40], sex [41], BMI [42], daily physical activity [43], presence of AF [44], presence of sarcopenia [45], presence of anemia [46], epicardial adipose tissue thickness [47], medication of β-blocker [48], and presence of T2DM. These independent variables have been reported to be associated with exercise capacity in patients with HFpEF.

## Results

### Selection of study participants

Among all 149 study participants, 6% were obese, 58.4% were overweight, 9.4% were current smokers, 55% had dyslipidemia, and 52.3% had hypertension. The study participants included 50 controls (2% with obesity, 62% with overweight, 58% with dyslipidemia, and 22% with hypertension), 48 had HFpEF without T2DM (14.6% with obesity, 47.9% being overweight, 52.1% with dyslipidemia, 62.5% with hypertension, old myocardial infarction (OMI) with 17%, AF with 65%, NYHA functional classification III with 42%), and 51 had HFpEF with T2DM (2% with obesity, 64.7% being overweight, 54.9% with dyslipidemia, and 72.5% with hypertension, OMI with 35%, AF with 55%, NYHA functional classification III with 55%). The median duration of diabetes in the HFpEF-with-T2DM group was 9.3 years (Table 1).

**Table 1:** Clinical characteristics of all the groups

Characteristics	Control (n = 50)	HFpEF without T2DM (n = 48)	HFpEF with T2DM (n = 51)	P-value
HFA-PEFF score		5 (IQR, 5–6)*	5 (IQR, 5–6)*	< 0.001
H_2_FPEF score		6 (IQR, 4–7)*	6 (IQR, 4–7)*	< 0.001
NYHA functional classification				
Class II	0	58	45	0.188
Class III	0	42	55	0.188
Comorbidities				
Old myocardial infarction (%)	0	19	35	0.065
Atrial fibrillation (%)	0	65	55	0.443
Anemia (%)	6	13*	24*^, †^	0.038
Hypertension (%)	22	63*	73*	< 0.001
Dyslipidemia (%)	58	52	55	0.841
Obesity (%)	2	15*	2	0.011
Overweight (%)	62	48	65	0.195
Sarcopenia (%)	4	4	20*^, †^	0.009
Diabetic duration (years)	0	0	9.3 (IQR, 8.8–9.7)*^, †^	< 0.001
Age (years)	75 (IQR, 72–78)	74 (IQR, 70–78)	74 (IQR, 72–77)	0.804
Male (%)	50	48	49	0.979
Anthropometric parameters				
Height (cm)	163 (IQR, 159–169)	163 (IQR, 154–171)	162 (IQR, 155–169)	0.515
Weight (kg)	70 (IQR, 66–72)	69 (IQR, 63–74)	66 (IQR, 62–73)	0.380
Body mass index (kg/m^2^)	26.1 ± 1.9	26.3 ± 3.0	25.9 ± 2.1	0.738
Body surface area (m^2^)	1.75 ± 0.11	1.75 ± 0.15	1.72 ± 0.14	0.442
Waist circumference (cm)	103 (IQR, 99–105)	116 (IQR, 113–125)*	116 (IQR, 112–123)*	< 0.001
Physical activity				
Steps (steps/days)	7603 (IQR, 6,602–8,430)	4695 (IQR, 3,774–5,276)*	4977 (IQR, 4,648–5,911)*	< 0.001
Movement related to calorie consumption (kcal/days)	294 (IQR, 253–338)	180 (IQR, 149–209)*	201 (IQR, 165–230)*	< 0.001
Components of sarcopenia				
Appendicular skeletal muscle index (kg/m^2^)	7.3 ± 0.9	7.1 ± 0.8	6.9 ± 0.9*	0.038
Hand grip (kg)	26.4 (IQR, 20.5–29.5)	22.4 (IQR, 19.7–28.8)	21.7 (IQR, 19.9–25.1)*	0.007
Sit to stand-5 (s)	7.2 (IQR, 6.8–7.9)	8.9 (IQR, 8.2–9.5)*	8.8 (IQR, 8.3–10.2)*	< 0.001
Preference and medication				
Smoker (%)	24	27	24	0.907
Angiotensin-converting-enzyme inhibitor (%)	0	60*	69*	< 0.001
Angiotensin II Receptor Blocker (%)	4	46*	59*	< 0.001
β blocker (%)	0	60*	65*	< 0.001
Calcium-channel blocker (%)	0	38*	57*	< 0.001
Diuretic (%)	0	6	4	0.220
Statin (%)	34	98*	84*	< 0.001
Fibrate (%)	10	0	4	0.062
Ezetimibe (%)	22	40*	76*	< 0.001
Biguanide (%)	0	0	78*^, †^	< 0.001
Sulphonylurea (%)	0	0	69*^, †^	< 0.001
α-glucosidase inhibitor (%)	0	0	20*^, †^	< 0.001
Sodium glucose cotransporter-2 inhibitor (%)	0	0	25*^, †^	< 0.001
Dipeptidyl peptidase-4 inhibitor (%)	0	0	22*^, †^	< 0.001
Biochemical analysis and blood pressure				
Total Cholesterol (mg/dL)	224 (IQR, 211–232)	226 (IQR, 215–232)	220 (IQR, 213–229)	0.741
Low–density lipoprotein cholesterol (mg/dL)	124 (IQR, 117–131)	142 (IQR, 134–151)*	140 (IQR, 133–144)*	< 0.001
High–density lipoprotein cholesterol (mg/dL)	58 (IQR, 54–62)	51 (IQR, 43–55)*	51 (IQR, 44–56)*	< 0.001
Triglyceride (mg/dL)	130 (IQR, 117–142)	152 (IQR, 141–168)*	152 (IQR, 142–171)*	< 0.001
Hemoglobin A1c (%)	5.6 (IQR, 5.4–5.7)	5.2 (IQR, 4.9–5.4)*	9.7 (IQR, 9.1–10.2)*^, †^	< 0.001
Fasting plasma glucose (mg/dL)	118 (IQR, 102–122)	98 (IQR, 93–105)*	159 (IQR, 149–168)*^, †^	< 0.001
HOMA-IR (%)	1.7 (IQR, 1.6–2.1)	1.3 (IQR, 1.1–1.5)*	3.5 (IQR, 3.0–3.8)*^, †^	< 0.001
eGFR at cystatin C (mL/min/1.73m^2^)	71 (IQR, 70–78)	58 (IQR, 55–63)*	50 (IQR, 47–54)*^, †^	< 0.001
Brain natriuretic peptide (pg/mL)	18 (IQR, 17–21)	173 (IQR, 148–209) *	202 (IQR, 173–219)*	< 0.001
Hemoglobin (g/dL)	14.0 (IQR, 13.4–14.4)	13.3 (IQR, 12.8–13.8)*	12.4 (IQR, 12.1–13.1)*^, †^	< 0.001
Systolic Blood Pressure (mmHg)	126 (IQR, 122–128)	142 (IQR, 124–148)*	142 (IQR, 128–148)*	< 0.001
Diastolic Blood Pressure (mmHg)	72 (IQR, 66–75)	68 (IQR, 66–75)	66 (IQR, 63–72)	0.067

Normal distribution data are expressed as means ± standard deviations, non-normal distribution data are expressed as medians, and nominal variables are expressed as percentages.

*HFpEF* heart failure with preserved ejection fraction, *IQR* interquartile range, *NYHA* New York Heart Association, *HOMA-IR* homeostasis model assessment of insulin resistance, *eGFR* estimated glomerular filtration rate

^*^P < 0.05 vs the Control group

^†^P < 0.05 vs the HFpEF-without-T2DM group

### Clinical characteristics among the three groups

Age, sex, BMI, BSA, percentage of overweight, dyslipidemia carriers, and current smoker were not significantly different between the three groups. Daily physical activity, sit-to-stand-five, medications, and lipid metabolism indicators were significantly worse in patients with HFpEF compared with the control group. Glucose metabolism indicators, such as hemoglobin A1c and fasting plasma glucose test, glomerular filtration rate for renal function, and the prevalence of anemia and sarcopenia were significantly worse in the HFpEF-with-T2DM group than in the HFpEF-without-T2DM group. The BNP tended to be higher in the HFpEF-with-T2DM group; however, there was no significant difference between the with- or without-T2DM groups (Table 1).

### Echocardiography data among the three groups

There were no significant differences between the three groups in LV end-diastolic diameter and LV end-diastolic volume. The epicardial adipose tissue was thicker in the HFpEF group than in the control group and was thicker in the HFpEF-with-T2DM group than in the HFpEF-without-T2DM group. LV structural and functional parameters, LV inflow parameters, and LV-GLS were significantly worse in the HFpEF group than in the control group, but there was no significant difference between the HFpEF-with-T2DM group and the HFpEF without T2DM group. The median LVEF for all groups was > 60%. Furthermore, there was no significant difference in the LA volume index in the HFpEF with or without T2DM groups, but LA emptying fraction and LA-GLS were significantly worsened in the HFpEF-with-T2DM group (Table 2).

**Table 2:** Echocardiography data of all the groups

Characteristics	Control (n = 50)	HFpEF without T2DM (n = 48)	HFpEF with T2DM (n = 51)	P-value
Epicardial adipose tissue thickness (mm)	5.1 (IQR, 4.3–6.8)	7.9 (IQR, 7.4–8.3)*	8.8 (IQR, 8.6–8.9)*^, †^	< 0.001
Interventricular septal thickness at end diastole (mm)	7.5 (IQR, 6.6–8.2)	10.2 (IQR, 9.3–10.6)*	9.8 (IQR, 9.3–10.8)*	< 0.001
Posterior wall thickness at end diastole (mm)	7.6 (IQR, 6.6–8.3)	10.2 (IQR, 9.4–10.6)*	9.9 (IQR, 9.3–10.7)*	< 0.001
Left ventricular end-diastolic diameter (mm)	46.5 ± 1.8	45.8 ± 2.5	46.1 ± 2.6	0.304
Left ventricular end-systolic diameter (mm)	27.0 (IQR, 26.1–28.3)	29.5 (IQR, 28.0–31.4)*	28.5 (IQR, 26.9–30.6)*	< 0.001
Left ventricular end-diastolic volume index (mL/m^2^)	57.4 ± 5.9	55.5 ± 7.5	57.4 ± 8.4	0.321
Left ventricular end-systolic volume index (mL/m^2^)	15.4 (IQR, 13.7–17.9)	19.6 (IQR, 16.6–22.5)*	18.5 (IQR, 16.2–22.2)*	< 0.001
Left ventricular ejection fraction (%)	74 (IQR, 67–77)	64 (IQR, 59–69)*	66 (IQR, 62–72)*	< 0.001
Left atrial ejection fraction (%)	58 (IQR, 57–61)	48 (IQR, 45–52)*	44 (IQR, 42–48)*^, †^	< 0.001
SI (mL/m^2^)	41.3 ± 6.3	35.7 ± 6.6*	38.4 ± 7.8	< 0.001
CI (L/min/m^2^)	2.9 ± 0.5	2.5 ± 0.5*	2.6 ± 0.6*	< 0.001
Left ventricular mass index (g/m^2^)	79 (IQR, 70–90)	116 (IQR, 104–125)*	119 (IQR, 102–130)*	< 0.001
Left atrial volume index (mL/m^2^)	29 (IQR, 28–30)	36 (IQR, 35–40)*	35 (IQR, 33–38)*	< 0.001
Relative wall thickness	0.32 (IQR, 0.29–0.36)	0.44 (IQR, 0.42–0.47)*	0.43 (IQR, 0.41–0.47)*	< 0.001
E (cm/s)	89.4 (IQR, 80.1–91.4)	57.6 (IQR, 51.9–62.8)*	59.8 (IQR, 53.1–66.4)*	< 0.001
A (cm/sec)	83.9 (IQR, 80.0–89.2)	77.9 (IQR, 70.0–82.0)*	79.2 (IQR, 69.6–88.2)*	< 0.001
E/A	1.02 (IQR, 1.00–1.06)	0.79 (IQR, 0.71–0.86)*	0.81 (IQR, 0.70–0.93)*	< 0.001
DcT (cm/s)	195 (IQR, 186–218)	238 (IQR, 224–268)*	229 (IQR, 218–249)*	< 0.001
Lateral e′ (cm/s)	10.9 (IQR, 10.7–11.2)	5.2 (IQR, 3.9–6.2)*	5.4 (IQR, 4.3–6.1)*	< 0.001
Medial e′ (cm/s)	8.7 (IQR, 6.7–10.0)	2.8 (IQR, 2.4–3.6)*	2.8 (IQR, 2.5–3.7)*	< 0.001
Mean e′ (cm/s)	9.7 (IQR, 8.7–10.4)	3.9 (IQR, 3.2–4.9)*	4.1 (IQR, 3.4–5.0)*	< 0.001
E/e′ (cm/s)	9.0 (IQR, 8.3–9.6)	14.6 (IQR, 13.2–16.6)*	14.7 (IQR, 13.7–16.0)*	< 0.001
Peak tricuspid regurgitation velocity (m/s)	2.2 (IQR, 2.1–2.3)	2.9 (IQR, 2.6–3.1)*	2.9 (IQR, 2.8–3.0)*	< 0.001
Left ventricular global longitudinal strain (%)	−22.0 (IQR, −18.9– −23.2)	−15.0 (IQR, −16.4– −14.3)*	−14.9 (IQR, −16.1– −13.9)*	< 0.001
Left atrial global longitudinal strain (%)	35.0 (IQR, 32.8–38.8)	31.9 (IQR, 30.5–33.8)*	27.6 (IQR, 25.5–30.5)*^, †^	< 0.001
Mitral regurgitation				
Mitral regurgitation volume (mL)	–	30.6 (IQR, 12.3–31.4)	30.5 (IQR, 16.4–31.3)	0.807
Effective regurgitant orifice area (cm^2^)	–	0.23 (IQR, 0.10–0.24)	0.22 (IQR, 0.14–0.27)	0.272
Mild (%)	–	21	25	0.583
Moderate (%)	–	25	29	0.622
Estimated pulmonary artery systolic pressure (mmHg)	29.3 (IQR, 27.4–32.6)	43.1 (IQR, 40.4–48.4)*	42.0 (IQR, 37.5–48.2)*	< 0.001
Presence of concentric remodeling (%)	0	77*	75*	< 0.001
Presence of eccentric hypertrophy (%)	0	73*	82*	< 0.001
Presence of concentric hypertrophy (%)	0	50*	57*	< 0.001

Normal distribution data are expressed as means ± standard deviations, non-normal distribution data are expressed as medians, and nominal variables are expressed as percentages.

* HFpEF* heart failure with preserved ejection fraction, *IQR* interquartile range, *SI* stroke volume index, *CI* cardiac output index, *E* peak early flow velocity, *A* Late diastolic flow velocity, *E/A* ratio of peak early and late diastolic flow velocities, *DcT* deceleration time, *e′* peak early diastolic tissue velocity, *E/e'* ratio of the mitral inflow early diastolic velocity to the mean e′ velocity from the septal and lateral sides of the mitral annulus

^*^P < 0.05 vs the Control group

^†^P < 0.05 vs the HFpEF-without-T2DM group

### CPET and hemodynamic data

**Fig. 1:**
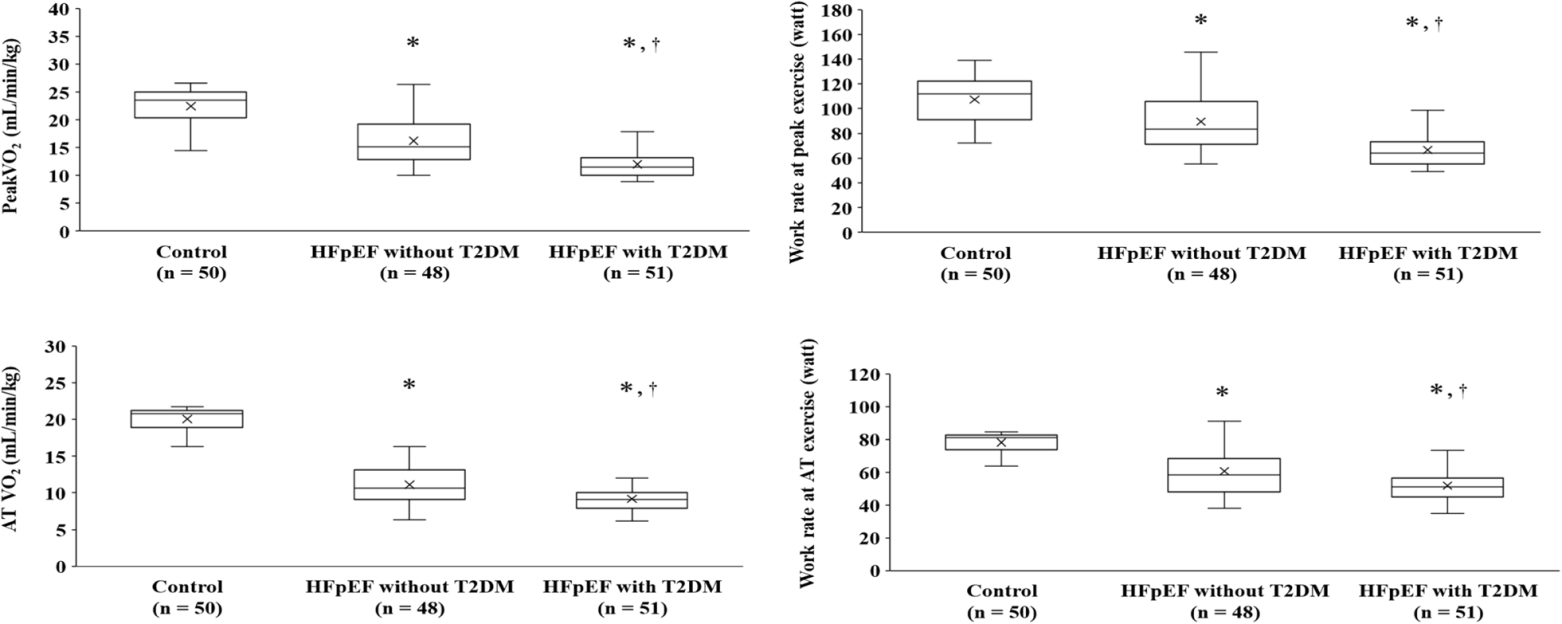
Exercise capacity and work rate data of all groups. Analysis of covariance was adjusted for the presence or absence of sarcopenia as well as hemoglobin level. *P < 0.05 vs the Control group, †P < 0.05 vs the HFpEF-without-T2DM group. *HFpEF* heart failure with preserved ejection fraction, *T2DM* type 2 diabetes mellitus, *peakVO*_*2*_ peak oxygen uptake, *AT VO*_*2*_ oxygen uptake at anaerobic threshold

**Fig. 2:**
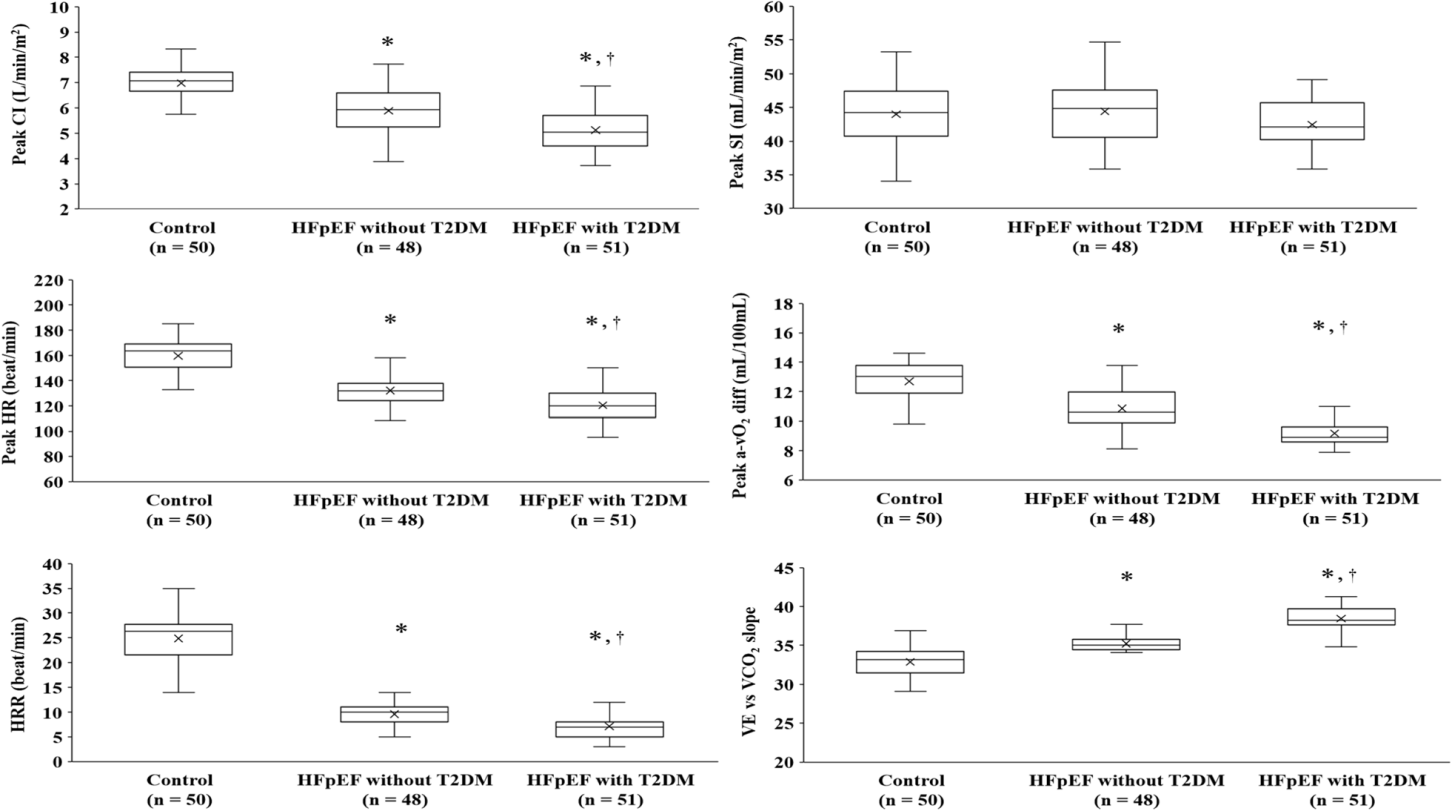
Hemodynamics data of all groups. Analysis of covariance was adjusted for medication of β-blocker as well as hemoglobin level. *P < 0.05 vs the Control group, †P < 0.05 vs the HFpEF-without-T2DM group. *HFpEF* heart failure with preserved ejection fraction, *T2DM* type 2 diabetes mellitus, *CI* cardiac output index, *SI* stroke volume index, *HR* heart rate, *a-vO*_*2*_ diff Arterial-venous oxygen difference, *HRR* heart rate recovery, *VE* vs *VCO*_*2*_* slope* ventilatory equivalent versus carbon dioxide output slope

Regarding CPET data, the median peak respiratory exchange ratios were > 1.10 in all groups, and a no-load shortage was observed. The highest peakVO_2_ value, peak watt, ATVO_2_, and AT watt were observed in the control group, followed by the HFpEF-without-T2DM group and the HFpEF-with-T2DM group (Fig. 1, Additional file 4). The highest CI, HR, and a-vO_2_ diff values were also observed in the control group, followed by the HFpEF-without-T2DM and HFpEF-with-T2DM groups. However, the peak SI was not significantly different among the three groups (Fig. 2, Additional file 5). The highest prevalence of chronotropic incompetence and the abnormality of HRR were observed in the HFpEF-with-T2DM group, followed by the HFpEF-without-T2DM group, and the control group (Additional file 2). The highest VE vs VCO_2_ slope, an index of ventilation efficiency during exercise, was observed in the HFpEF-with-T2DM group, followed by the HFpEF-without-T2DM group and the control group (Fig. 2, Additional file 5).

### ***Associations between T2DM and peakVO***_***2***_*** in patients with HFpEF***

In the multiple linear regression analysis with the peakVO_2_ as the dependent variable, age, sex, BMI, presence of AF, and T2DM (*β* = −0.551, 95% confidence interval = −5.597– −3.200, P < 0.001) were found to be the independent factors associated with the peakVO_2_ (*R*^*2*^ = 0.476) (Table 3).

**Table 3:** Multiple linear regression analysis with peak oxygen uptake as the dependent variable

Independent variables	Peak oxygen uptake						
	R^2^	Standard error	Standardized β	95% confidence interval		Variance Inflation Factor	P-value
	0.476						
Age		0.069	−0.203	−0.322	−0.048	1.008	0.008
Sex		0.610	−0.185	−2.686	−0.265	1.034	0.017
Body mass index		0.120	−0.214	−0.574	−0.098	1.033	0.006
Treatment with β-blocker							
Steps (daily physical activity)							
Presence of atrial fibrillation		0.611	−0.304	−3.673	−1.247	1.008	< 0.0001
Presence of sarcopenia							
Hemoglobin level							
Epicardial adipose tissue thickness							
Presence of Type 2 diabetes mellitus		0.604	−0.551	−5.597	−3.200	1.013	< 0.0001

Multiple linear regression analysis was performed using the stepwise method; the dependent variables were peak oxygen uptake. We selected as independent variables known parameters that were found to be significantly associated with peak oxygen uptake in patients with HFpEF [40–48]. To confirm multicollinearity between the independent variables, a correlation coefficient of  ≥ 0.8 or a variance inflation factor of  ≥ 5.0 was looked for, but neither was confirmed. In addition, on performing the Shapiro–Wilk test on residuals, the significance probability was 0.112, thus, confirming their normal distribution

## Discussion

This study had four major findings involving patients with HFpEF and T2DM diagnosed according to stringent criteria. First, patients with HFpEF had a higher prevalence of anemia and sarcopenia and significantly worsened BNP and renal function than age-, sex-, and BMI-matched control groups, and the coexistence of T2DM further significantly deteriorated these indices. Second, the HFpEF-with-T2DM group had the worst LA function among the three groups. Third, patients with HFpEF had lower objective exercise capacity than the control group, and the coexistence of T2DM further significantly deteriorated exercise intolerance. Furthermore, although there was no significant difference in the peak SI among the three groups, the peak CI, HR, and a-vO_2_ diff values were significantly lower, and VE vs VCO_2_ slope was significantly higher in patients with HFpEF, and coexistence of T2DM further deteriorated the hemodynamic response during exercise. Finally, T2DM comorbidity was independently associated with peakVO_2_ in patients with HFpEF, even after multivariate adjustment. These results suggest the possibility of identifying characteristic pathophysiology that contributes to reduced exercise capacity in patients with HFpEF with T2DM and intervention pathways for its improvement.

### Clinical characteristics in patients with HFpEF with T2DM

Our results showed that the estimated glomerular filtration rate and the prevalence of anemia were significantly worse in the HFpEF-with-T2DM group. A finding supported in a similar study by Lindman et al. [11]. However, although there was no significant difference in BMI among the three groups, the prevalence of obesity increased significantly in the HFpEF-without-T2DM group; this contradicts our hypothesis and the Lindman et al. report. Although the reason for this is unclear, it is known that East Asians, such as the Japanese, develop T2DM even when their BMI is < 25 kg/m^2^ [49]; this is often associated with skeletal muscle dysfunction and sarcopenia, involving major organs that consume glucose [50]. This report partially supports our data showing a significant increase in the prevalence of sarcopenia in the HFpEF-with-T2DM group. Although our study is the first to clarify the prevalence of sarcopenia according to the presence or absence of T2DM in East Asian patients with HFpEF, owing to the relatively small number of samples, further large-scale multicenter studies are needed in the future.

### LV and LA structural and functional data in patients with HFpEF with T2DM

From the results of our study, LV structure and function tended to partially worsen in the HFpEF-with-T2DM group, such as LV filling pressure, but there was no significant difference between the with or without T2DM groups. This result is consistent with the report by Lindman et al. [11]. The current study suggests that the comorbidity of T2DM in patients with HFpEF is unlikely to significantly affect LV structure and functions. This issue needs further investigation in a large multicenter study with an increased sample size. Furthermore, as reported by Wehner et al. [51], HF with LVEF ≥ 65% observed in most of our study participants is an HF phenotype of concern for life prognosis. In this large cohort study of 203,135 individuals, the group with LVEF 60–65% had the lowest mortality, while those with LVEF < or > 60–65% had a higher risk of death. Furthermore, even after adjusting for multiple confounders, the LVEF ≥ 70% group was associated with higher mortality in inpatient and outpatient settings. Wehner et al. defined heart failure with LVEF ≥ 65% as heart failure with supra-normal LVEF [51]. In this study, we found a U-shaped relationship between mortality and LVEF, suggesting that it may be inappropriate. The increased mortality in the LVEF ≥ 65% group has been shown to persist even after adjusting for other complications that may increase LVEF, such as MR, LV hypertrophy, and anemia. In our study results (as shown in the Additional file 3), the peakVO_2_, which is one of the life prognostic factors in patients with HF, showed the maximum value in the range of LVEF 60.1–65.0. However, those with higher LVEF showed a significantly lower value. This result suggests that it may partially explain the higher mortality in the population with LVEF ≥ 65%. As our study was a cross-sectional study, we were unable to explain the mechanism of this phenomenon. However, further studies are needed to elucidate the precise pathophysiology and characteristics of this phenotype with high non-cardiovascular mortality. In contrast, LA structure and function worsened significantly in the HFpEF-with-T2DM group. There is one report on LA function and life prognosis in patients with HFpEF with T2DM, but the median age of the study participants was approximately 60 years, which is significantly younger than that for patients with HFpEF. Thus, the results cannot be generalized [52]. To the best of our knowledge, our study is the first to compare the LA structure and function in the presence or absence of T2DM in patients with HFpEF with a median age of 74 years. Worse LA function is independently associated with exercise intolerance in patients with T2DM and a significantly higher risk of heart failure-related hospitalization in patients with HFpEF [53, 54]. Therefore, the results on LA structure and function obtained in this study suggest that it may be a factor in explaining the worse clinical outcome of patients with HFpEF with T2DM [11].

### Exercise capacity and hemodynamics in patients with HFpEF with T2DM

The peakVO_2_ objectively evaluated by CPET in patients with HFpEF is one of the indicators closely related to life prognosis [55]. Patients with HFpEF had lower peakVO_2_ than age-matched controls, and HFpEF with T2DM has been reported to further reduce peakVO_2_ [9, 11]. However, the determinants of exercise intolerance in patients with HFpEF with T2DM have not been investigated. Our study extended this knowledge by evaluating hemodynamics during submaximal exercise in HFpEF with T2DM.

The peak SI, one of the hemodynamic indices, showed no significant difference among the three groups; this is consistent with the findings of Haykowsky et al. and Bhella et al. in age-matched patients with HFpEF [56, 57]. However, a study by Borlaug et al. reported that the peak SV was significantly decreased in the HFpEF group, which is paradoxical to our findings [58]. The definitive reason for this is unclear, but we performed CPET in an upright position, whereas Borlaug et al. reported that the posture during CPET was supine [58]. Differences in posture affect preload during exercise. Exercise in the supine position increased preload compared to at rest, which corresponds to the flat portion of the Frank–Starling relationship. In the study by Borlaug et al., there was little change in his SV index at rest and during maximal exercise (SI at rest = 40 mL/m^2^, peak exercise: SI = 47 mL/m^2^). In our study, the SV index did not increase with AT intensity or higher but increased appropriately with resting sitting position to peak exercise.

Although there was no significant difference in the peak SI, central and peripheral factors, such as peak CI, HR, and a-vO_2_ diff values, decreased in the HFpEF-with-T2DM group; this may be related to the prevalence of chronotropic incompetence, abnormality of HRR, and presence of sarcopenia. Although there was no significant difference in resting HR among the three groups, the HR response flattened out with increasing exercise load in the HFpEF group, especially in the HFpEF-with-T2DM group. SV reached a plateau at 40–50% of maximal exercise, after which an increase in HR led to a rise in CO [59]. A higher prevalence of chronotropic incompetence was present in the HFpEF-with-T2DM group [11]. This report partially supports our findings. However, although HR response is one of the factors of exercise intolerance, it cannot be concluded as a determinant of exercise intolerance as this study is a case–control study.

Furthermore, in the HFpEF-with-T2DM group, approximately 30% of patients terminated their CPET because of decreased pedal velocity and the prevalence of sarcopenia. Therefore, even if the peak respiratory exchange ratio exceeds 1.1, early termination of exercise due to muscle weakness in the lower extremities cannot be ignored. Therefore, further investigation considering these confounding factors is required.

Peak a-vO_2_ diff was significantly lower in the HFpEF-with-T2DM group. Our results showed that exercise intolerance in HFpEF with T2DM is closely associated with a reduced oxygen extraction capacity of peripheral tissues. Decreased peak a-vO_2_ diff has been implicated as a significant cause of exercise intolerance in patients with HFpEF and T2DM [55, 60]. These reports support some of our findings. In particular, sarcopenia, one of the non-cardiac factors, appears to be closely associated with exercise intolerance in patients with HFpEF [45]. Nesti et al. reported hemoglobin as a predictor of the a-vO_2_ diff [10]. In our study, the HFpEF-with-T2DM group also showed a significant increase in the prevalence of anemia. Multiple reports and our results suggest that extracardiac factors may be closely related to exercise intolerance in a cohort characterized by HFpEF with T2DM. However, it should be noted that in our study, the a-vO_2_ diff was measured as an estimate calculated using the Fick equation. Furthermore, the prevalence of sarcopenia was significantly increased in the HFpEF-with-T2DM group, but the differences in its constituent factors (e.g., appendicular skeletal muscle index, hand grip, and the five-time chair-stand test as a physical function) were slight. Therefore, it cannot be concluded that peripheral factors, such as peak a-vO_2_ diff, are determinants of exercise capacity in patients with HFpEF with T2DM and should be left to the influential hypothesis stage.

VE vs VCO_2_ slope, an index of ventilation efficiency during exercise, was higher in the HFpEF-with-T2DM group than in the HFpEF-without-T2DM group. When a pulmonary disease is excluded, as in our study, VE vs VCO_2_ slope is an indicator of pulmonary artery blood flow and ventilation/perfusion imbalance, and high values in patients with HFpEF have been reported to be associated with survival prognosis [60]. To the best of our knowledge, this is the first report on the ventilatory function of HFpEF with T2DM during exercise. Cardiac autonomic neuropathy may exacerbate the ventilatory response to exercise in patients with diabetes by excessively increasing the respiratory rate and alveolar ventilation [61]. In our study, as shown in Additional file 2, 84% of the patients in the HFpEF-with-T2DM group had abnormalities of HRR and cardiac autonomic neuropathy. Therefore, it cannot be denied that the presence of ventilatory/perfusion imbalance and cardiac autonomic neuropathy was associated with insufficient CO in the HFpEF-with-T2DM group caused an increase in VE vs VCO_2_ slope.

### T2DM as an independent factor of exercise intolerance in patients with HFpEF

Multiple regression analysis showed that T2DM was independently associated with peakVO_2_ in patients with HFpEF. T2DM has been reported as a predictor of peakVO_2_ regardless of LVEF [62]. Our study has clinical significance as we enrolled patients with HFpEF aged 65–80 years, who are likely to be encountered clinically, and presented results after adjusting for multiple confounding factors, such as sarcopenia and daily physical activity. Although the underlying cause of exercise intolerance in HFpEF is multifactorial, our results suggest that T2DM may adversely affect multiple predictors.

Furthermore, exercise intolerance in HFpEF with T2DM may be associated with chronotropic incompetence, decreased ventilation efficiency during exercise as central factors, and decreased a-vO_2_ diff as peripheral factors. Additionally, cardiac autonomic neuropathy, anemia, and sarcopenia may also have an effect. Therefore, as a suggestion for future interventions in cases of HFpEF with T2DM with poor prognosis, sodium-glucose cotransporter two inhibitors may improve glycemic control and anemia [63]. It has also been suggested that cardiac rehabilitation, as a non-pharmacological intervention, may improve cardiac autonomic neuropathy and sarcopenia. Further studies are needed to determine whether these interventions improve exercise intolerance and prognosis.

### Limitations

In this study, selection bias cannot be completely ruled out because it was a single-center study. Moreover, this study included only Japanese individuals, who differ from Caucasians in race and physique. A total of 48% of patients with HFpEF were classified as NYHA class III, but only 5% received diuretics at the time of our investigation. The BNP levels and peak tricuspid regurgitation velocity of patients with HFpEF in our study possibly indicated that many of those with NYHA class III may have experienced fluid retention, and that they were not receiving adequate medication at the initial visit. As noted in the guidelines, in patients with HF who have fluid retention, diuretics are recommended to relieve congestion, improve symptoms, and prevent worsening HF [64, 65]. Therefore, the impact of this on exercise capacity cannot be denied. Impedance cardiography, a noninvasive method for assessing CO, has been reported to be highly correlated with the direct Fick method in healthy individuals. However, SV may be overestimated when patients with HF are included as participants [66]. Therefore, errors may have occurred during measurement in participants with the same HF symptoms. Nevertheless, our study participants had a more preserved LVEF than the Kemps et al. study [66]; patients with dilated cardiomyopathy were excluded as their clinical characteristics were significantly different. A stress test that combines CPET and echocardiography shows a clinically acceptable measurement accuracy, consistent with Fick's CO value measured directly during exercise.

Further, various types of information can be obtained during exercise (e.g., LV-GLS, E/e', LVEF); this may provide a compatible alternative to the invasive direct Fick method [67]. The a-vO_2_ diff was also calculated from Fick's formula, and we cannot conclude that the decline in a-vO_2_ is a determinant of peakVO_2_ due to the methodological limitations of this study. Finally, we did not collect biomarker data other than the BNP levels. Obtaining biomarkers other than BNP, especially biomarkers of vasodilatation and fibrosis, such as endothelin and galectin, may provide suggestions for LA pathological changes and a-vO_2_ diff and knowledge that will help us better understand the mechanisms.

## Conclusions

The results of this case–control study based on patients with HFpEF diagnosed by the stringent criteria showed that T2DM was independently associated with peakVO_2_ in patients with HFpEF. Furthermore, HFpEF combined with T2DM may lead to additive decreases in exercise capacity, HR response, peripheral oxygen extraction, and ventilation efficiency. Our results suggest that patients with HFpEF with T2DM have a characteristic pathophysiology, such as cardiac autonomic neuropathy, anemia, and sarcopenia, and these factors may be related to peakVO_2_ determinants. Multiple factors cause exercise intolerance in patients with HFpEF with T2DM, but our findings may help identify intervention targets. Further investigation is needed through clinical trials based on large-scale pharmacological and non-pharmacological diabetes care interventions in this unique cohort population.

## Supplementary Information

**Additional file 1.**  Detailed information about Materials and Methods.

**Additional file 2: **Cardiopulmonary exercise testing and hemodynamics data of all groups

**Additional file 3: **Exercise capacity and ventilatory equivalent versus carbon dioxide output slope according to left ventricular ejection fraction. Analysis of covariance was adjusted for the presence or absence of sarcopenia as well as hemoglobin level. * P <0.05 vs 50.0-55.0 group, † P <0.05 vs 55.1-60.0 group, ‡ P <0.05 vs 65.1-70.0 group, § P <0.05 vs >70.1 group. Abbreviations: peakVO_2_, peak oxygen uptake; VE vs VCO_2_ slope, Ventilatory equivalent versus carbon dioxide output slope.

**Additional file 4: **Exercise capacity and work rate data of all groups. Analysis of covariance was adjusted for the presence or absence of sarcopenia as well as hemoglobin level. The HF groups consisted of 61 patients excluding H_2_FPEF score ≤5 and HFA-PEFF score ≤4. * P <0.05 vs the Control group, † P <0.05 vs the HFpEF without T2DM group.

**Additional file 5:** Hemodynamic data of all groups. Analysis of covariance was adjusted for medication of β-blocker as well as hemoglobin level. The HF groups consisted of 61 patients excluding H_2_FPEF score ≤5 and HFA-PEFF score ≤4. * P <0.05 vs the Control group, † P <0.05 vs the HFpEF without T2DM group. HFpEF, heart failure with preserved ejection fraction; T2DM, type 2 diabetes mellitus; CI, cardiac output index; SI, stroke volume index; HR, heart rate; a-vO_2_ diff, Arterial-venous oxygen difference; HRR, heart rate recovery; VE vs VCO_2_ slope, Ventilatory equivalent versus carbon dioxide output slope.

## Data Availability

The datasets during and/or analyzed during the current study are available from the corresponding author upon reasonable request.

## Abbreviations

AF: Atrial fibrillation
BMI: Body mass index
BSA: Body surface area
CI: Cardiac output index
peak CI: Peak cardiac output index
CPET: Cardiopulmonary exercise testing
HF: Heart failure
HFpEF: HF with preserved ejection fraction
HOMA-IR: Homeostasis model assessment of insulin resistance
SV: Stroke volume
LVEF: Left ventricular ejection fraction
VO_2_: Oxygen uptake
peakVO_2_: Peak oxygen uptake
AT: Anaerobic threshold
VE vs VCO_2_ slope: Ventilatory equivalent versus carbon dioxide output slope
HRR: Heart rate recovery
HR: Heart rate
CO: Cardiac output
a-vO_2_ diff: Arteriovenous oxygen difference
LV: Left ventricular
LVH: LV hypertrophy
LA: Left atrial
LAV: LA volume
WHO: World Health Organization
BNP: Brain natriuretic peptide
RWT: Relative wall thickness
GLS: Global longitudinal strain
SI: Stroke volume index
OMI: Old myocardial infarction
NYHA: New York Heart Association
