# Supplementary material for: Association of complication of type 2 diabetes mellitus with hemodynamics and exercise capacity in patients with heart failure with preserved ejection fraction: a case–control study in individuals aged 65–80 years
Source: Cardiovasc Diabetol. 2023 Apr 28;22:97. doi: 10.1186/s12933-023-01835-2 (PMC10148403; doi:10.1186/s12933-023-01835-2)
Supplement: Supplementary file 1 — Additional file 1. Detailed information about Materials and Methods. [file 12933_2023_1835_MOESM1_ESM.docx]

**Additional File 1**

BMI (kg/m^2^) = body weight (kg) / height^2^ (m)

BSA (m^2^) = body weight^0.425^ × height^0.725^ × 0.007184.

eGFR (mL/min/1.73 m^2^) = [104 × cystatin-c^-1.019^ × 0.996^age^ × 0.929 (if female)] − 8

HOMA-IR = fasting insulin (μU/mL) × fasting glucose (mg/dL)/405.

SV (mL) = LVEDV (mL) – LVESV (mL)

LVEF (%) = SV / LVEDV (mL) × 100

LAVI (mL/m^2^) = LAV max / BSA (m^2^)

LAEF (%) = (LAV max – LAV min) / LAV max × 100

Estimated pulmonary artery systolic pressure (mmHg) = 4 × (tricuspid regurgitation velocity)^2^ + estimated right atrium pressure

HRR = peak heart rate or HR – HR 1 min after the end of exercise load

VO_2_/HR (mL/beat) = VO_2_ (mL) / HR (beats/min)

CO (L/min) = SV (mL/min) × HR (beats/min)

a-vO_2_ (mL/100 mL) = CO (L) / VO_2_ (mL)

**Materials and Methods**

***Study design and participants***

The exclusion criteria for participants in this study were as follows: exacerbation of subjective symptoms of heart failure (including dyspnea, and fatigue) within the past week, unstable angina or low threshold (induced by slow walking on level ground, two metabolic equivalents); severe valvular disease, for which surgery is indicated (especially aortic stenosis); severe left ventricular outflow tract stenosis (obstructive hypertrophic cardiomyopathy); untreated exercise-induced severe arrhythmia (ventricular fibrillation, persistent ventricular tachycardia); active myocarditis; acute systemic disease or fever; other diseases, for which exercise therapy is contraindicated (including moderate or higher aortic aneurysm, severe hypertension, thrombophlebitis, embolism within two weeks, and serious other organ damage); congenital cardiovascular disease; renal failure with estimated glomerular filtration rate <30; diagnosis of psychiatric disorders; malignant tumor; New York Heart Association class I and IV; and patients under 65 or over 80 years of age. From a total of 178 patients, 29 were excluded from the analysis (three did not agree with the explanation of the study, eight had a daily physical activity of either less than 500 steps or more than 20,000 steps, 13 lacked data on physical activity, and five were hospitalized due to orthopedic disease).

***Diagnosis of with heart failure with preserved ejection fraction (HFpEF)***

HFpEF was defined as the presence of clinical symptoms of heart failure (HF) (clinical HF symptoms were defined as subjective symptoms such as shortness of breath during exertion, orthopnea, and paroxysmal nocturnal dyspnea), a left ventricular ejection fraction >50%, and the presence of left ventricular diastolic dysfunction as defined by the American Society of Echocardiography/European Association of Cardiovascular Imaging (i.e., mean E/e′ >14, septal e′ <7 cm/s or lateral e′ <10 cm/s, tricuspid regurgitant velocity >2.8 m/s, left atrial volume index >34 mL/m^2^, where the above three points are considered satisfactory) [15]. In addition, we calculated the H_2_FPEF and HFA-PEFF scores to make the diagnosis of HFpEF more accurate [16, 17]. The H_2_FPEF score included the following six variables [16]: BMI >30 kg/m^2^, treatment with two or more antihypertensive medicines, paroxysmal or persistent AF, estimated pulmonary artery systolic pressure >35 mmHg, E/e' >9 by Doppler echocardiography, and age >60 years. These six variables were summed to give a score ranging from 0 to 9 points. The interpretation was as follows: 0 or 1 points, excludes diagnosis of HFpEF; 2–5 points, requires further testing to confirm the diagnosis of HFpEF; 6–9 points, probable HFpEF diagnoses. The scoring criteria are provided in Additional File 3. The definitions adopted for the HFA-PEFF criteria follow the HFA-PEFF diagnostic algorithm for HFpEF in a consensus report proposed in 2019 by the Heart Failure Association of the European Society of Cardiology [17]. The HFA-PEFF score includes echocardiographic screening for functional or morphological abnormalities and measurement of brain natriuretic peptide levels. In brief, 2 points are awarded if each major criterion is met and 1 point if the sub-criteria are met. If the total HFA-PEFF score is <1 point, the diagnosis of HFpEF is unlikely and investigation of alternative causes of the disease is warranted. Immediate diagnosis of HFpEF is recommended if the score is ≥5 points.

***Diagnosis of Type 2 diabetes mellitus***

Type 2 diabetes mellitus was diagnosed when the fasting plasma glucose level was ≥126 mg/dL, the 2 h value for the 75 g oral glucose tolerance test was ≥200 mg/dL, the causal plasma glucose level was ≥200 mg/dL, and the hemoglobin A1c level was ≥6.5% [18].

***Measurements of anthropometric parameters, biochemical data, and blood pressure***

The body height was measured to the nearest 0.1 cm using a wall-mounted stadiometer (DC250; Tanita Co. Ltd., Tokyo, Japan). The body weight of barefoot patients was measured to the nearest 0.1 kg using calibrated electronic digital scales (DC250; Tanita).

Brain natriuretic peptide, triglyceride, total cholesterol, high-density lipoprotein cholesterol, fasting plasma glucose, and fasting plasma insulin levels were measured using an enzymatic method. The hemoglobin A1c level was determined using high-performance liquid chromatography. It is expressed in the National Glycohemoglobin Standardization Program unit. An enzyme solution (Qualijent TG and Choletest N HDL; Sekisui Medical Co., Ltd., Tokyo, Japan) was added to 2–3 μL of the sample. The mixture was heated at 37°C for 5 min, and the absorbance was measured to determine the concentration.

Hypertension and dyslipidemia were determined according to the Japanese Diagnosis Criteria as follows: (1) systolic blood pressure (SBP) ≥130 mmHg and/or diastolic blood pressure ≥85 mmHg; (2) Triglyceride levels ≥150 mg/dL; (3) and high-density lipoprotein cholesterol levels <40 mg/dL [24].

***Evaluation of pulmonary artery systolic pressure and mitral regurgitation***

The pulmonary artery systolic pressure was estimated from the peak flow velocity of tricuspid regurgitation and estimated right atrial pressure using the simplified Bernoulli equation. The right atrial pressure was estimated from the inferior vena cava diameter and respiratory variation. The normal right atrial pressure was set at 3 mmHg when the diameter of the inferior vena cava was <21 mm and sniff reduced the diameter by >50%. Especially, pressure of 15 mmHg was set for inferior vena cava diameters >21 mm with <50% sniff variation or <20% resting respiratory variation. If none of these conditions apply, its middle 8 mmHg was used as the estimated right atrial pressure [26].

For mitral regurgitation (MR), visual qualitative evaluation was first performed. Tracing was performed at the time when the regurgitant jet could be visualized to the maximum, a small thin central jet was considered mild MR, a jet covering >50% of the LA area was considered severe MR, and an intermediate jet area between mild and severe was considered to be moderate MR. For quantitative evaluation, MR was recorded by continuous wave Doppler, the maximum waveform was traced, and the effective regurgitant orifice area (EROA) and mitral regurgitant volume (MR volume) were automatically measured by the proximal isovelocity surface area method. The use of the proximal isovelocity surface area method to quantify the severity of MR is strongly recommended by the European Association of Cardiovascular Imaging [27]. The severity of MR was graded as “mild” (EROA <0.20 cm^2^ or <MR volume 30mL), “moderate” (EROA 0.20–0.39 cm^2^ or MR volume 30–59 mL), and “severe” (EROA ≥0.40 cm^2^ or MR volume ≥60 mL) [28]. In our study, presence of severe valvular disease requiring surgery was one of the exclusion criteria. All MR severity data presented in Table 2 are results based on quantitative evaluation.

***Speckle-tracking imaging***

After manually tracing the endocardial border and selecting the appropriate wall thickness, the software automatically identified six segments in each view and tracked the motion of the acoustic markers. For systolic function assessment, parameters assessed from myocardial deformation curves, including the peak strain (defined as the most significant negative value on the strain curve) and peak systolic and early diastolic strain rates, were averaged from all segments measured.

***Measurement of* the *epicardial adipose tissue thickness***

Two-dimensional guided M-mode echocardiography was performed using a 2.5 MHz transducer with the participants in the left lateral decubitus position. The parasternal long- and short-axis views were measured from the trailing edge to the leading edge of the end-systolic right ventricular free wall. This information was saved for offline analysis. Then, the mean values measured in the parasternal long- and short-axis views were calculated.

***Measurement of exercise capacity and hemodynamic response***

The peakVO_2_ and anaerobic threshold were determined using the ramp loading method with an increase of 10 W/min after a rest period of 4 min and warm-up at 0 W for 4 min. The highest oxygen uptake after more than 30 s of the exercise was the peakVO_2_.

The peakVO_2_ was defined as the highest VO_2_ during the exercise [34]. The anaerobic threshold was determined using the V-slope method described by Beaver et al. [35].

A respiratory analyzer (AE300S; Minato Medical Science Co., Ltd. Tokyo, Japan) was used in breath-by-breath mode to measure expiratory gas. The exhaled gas data were analyzed using an average of 10 respiratory cycles.

The number of revolutions of the pedal during bicycle movement was 60 revolutions/min (rpm). The HR was constantly monitored at rest, during exercise, and during recovery using an electrocardiogram monitor (ML4500; Fukudadenshi Co., Ltd. Tokyo, Japan). The endpoints of the bicycle movement were as follows: (1) leveling off of VO_2_, (2) decrease in SBP by 10 mmHg with an exercise load and SBP of 250 mmHg or more, (3) rate of perceived exertion of respiratory and rate of perceived exertion of the lower extremity >17, (4) a respiratory exchange ratio of >1.15, and (5) a pedal speed of <50 rpm (≥3 s). It was considered complete if at least one of these five conditions was met.

The chronotropic incompetence was determined to be <80% of the predicted maximum HR, according to the report of Laforgia et al. [36]. Based on the report of Cole et al., an abnormal value for the HRR was defined as a reduction of 12 beats/min or less from the heart rate at peak exercise [37].

***Measurement of physical activity***

To objectively assess physical activity, movement-related calorie consumption during physical activity and the number of steps taken was measured continuously for a week by a Lifecorder (Suzuken Co., Ltd. Nagoya, Japan) worn in the lumbar region of the study participants. Data were accepted when ≥75% of Lifecorder was attached per unit of time and rejected when ≥25% defects were confirmed due to non-attachment. The defect criterion was as follows: continuous non-wearing for ≥3 h out of 12 h (body movement level 0 was regarded as non-wearing).

***Diagnosis of sarcopenia***

The skeletal muscle index was calculated by dividing the limb skeletal muscle mass measured using bioelectrical impedance analysis (InBody720, BIOSPACE CO., Urbandale, IA, USA) by the square of height (m).

The grip strength was measured with the dominant arm using a Smedley-type grip strength (T.K.K.5401, Takei Scientific Instruments CO., Tokyo, Japan).

The five-time chair-stand test starts from a chair with a height of 40 cm. The time required to repeat five times as fast as possible was measured in 0.01-s units.
